# Characterization of the mitochondrial genome of *parupeneus indicus* (perciformes, mullidae)

**DOI:** 10.1080/23802359.2022.2075287

**Published:** 2022-05-12

**Authors:** Xiongbo He, Zhisen Luo, Murong Yi, Yunrong Yan

**Affiliations:** a College of Fisheries, Guangdong Ocean University, Zhanjiang, China; b Marine Resources Big Data Center of South China Sea, Southern Marine Science and Engineering Guangdong Laboratory (Zhanjiang), Zhanjiang, China; c Guangdong Provincial Engineering and Technology Research Center of Far Sea Fisheries Management and Fishing of South China Sea, Guangdong Ocean University, Zhanjiang, China

**Keywords:** mitochondrial genome, Mullidae, phylogenetic analysis

## Abstract

*Parupeneus indicus*, a species in the family Mullidae, inhabits the coastal and inner lagoon reefs of the Indian and Pacific oceans. The mitochondrial genome of *P. indicus* is 16,566 base pairs in length and contains 13 protein-coding genes (PCGs), 22 transfer RNA (tRNA) genes, two ribosomal RNA (rRNA) genes, and a D-loop control region. The overall base composition is 26.58% A, 24.83% T, 30.39% C, and 18.19% G, indicating an AT-rich profile (51.41%). Phylogenetic analysis based on the 13 PCGs revealed a close evolutionary relationship between *P. indicus* and *P. barberinus*. The data obtained in this study represent a valuable mitogenomic resource for population studies in the family Mullidae and will contribute to gaining a better understanding of the conservation genetics and environmental DNA of these fish.

The vertebrate family Mullidae comprises 101 valid species (Fricke et al. 2021), including 35 recognized species in the genus *Parupeneus*, among which, the Indian goatfish *Parupeneus indicus* (Shaw, 1803), is widely distributed in the Indian and Pacific oceans, wherein it inhabits shallow sandy or silty areas (seagrass substrata) of coastal and inner lagoon reefs (Froese and Pauly 2021). In this study, we conducted phylogenetic and evolutionary analyses of *Parupeneus* and Mullidae based on assembly of the complete mitochondrial genome of *P. indicus*.

*P. indicus* specimens were collected from Zhanjiang City, Guangdong Province, China (21.024°N, 109.707°E) and have been deposited at the College of Fisheries, Guangdong Ocean University, with voucher number GOU100526 (The person in charge of the collection: Zhisen Luo; email: yryan_gdou@163.com). Genomic DNA was extracted from the muscle tissue of *P. indicus* using a TIANamp Marine Animals DNA Kit (Tiangen Biotech, Beijing, China), and next-generation sequencing was performed using the Illumina HiSeq platform (Illumina, San Diego, CA) as previously described (Yi et al. 2019; He et al. 2020). We accordingly obtained a total of 75,318,562 clean reads of 11,207,760,394 base pairs (bp) in size, within which protein-coding (PCG), ribosomal RNA (rRNA), and transfer RNA (tRNA) genes were manually inspected following identification using MITOS (Bernt et al. 2013). The complete mitochondrial genome of *P. indicus* has a total length of 16,566 bp, with a T, C, A, and G composition of 24.83%, 30.39%, 26.58%, and 18.19%, respectively (GenBank accession number: OL581685), and contains a typical set of 13 PCGs, 22 tRNAs, two rRNAs (12S and 16S rRNAs), and a single D-loop control region. With the exception of COI, which uses a GTG start codon, all PCGs start with an ATG sequence. As stop codons, six PCGs (COI, COIII, ATP6, NAD3, NAD4L, and NAD5) use TAA, whereas TAG is used by ND1, ATP8, and ND6, and the remaining four PCGs (COII, ND2, ND4, and Cyt *b*) are terminated by an incomplete T stop codon (Figure 1). The 22 tRNA genes have lengths ranging from 67 (tRNA^Cys^) to 70 (tRNA^Lys^ and tRNA^Leu^) bp, all of which, with the exception of tRNA^Ser^ (AGY), lack a dihydrouridine arm. To determine the taxonomic position of *P*. *indicus*, we constructed a phylogenetic tree using the maximum likelihood approach with PhyloSuite (Zhang et al. 2020). Phylogenetic relationships determined based on analyses of a concatenated set of the 13 PCG sequences of eight mullid species indicate that evolutionarily, *P. indicus* is most closely related to *P. barberinus* (Figure 1). The valuable mitogenomic information obtained in this study will provide a valuable resource for further studies on the conservation genetics, environmental DNA, and populations of species in the family Mullidae.

**Figure 1.**
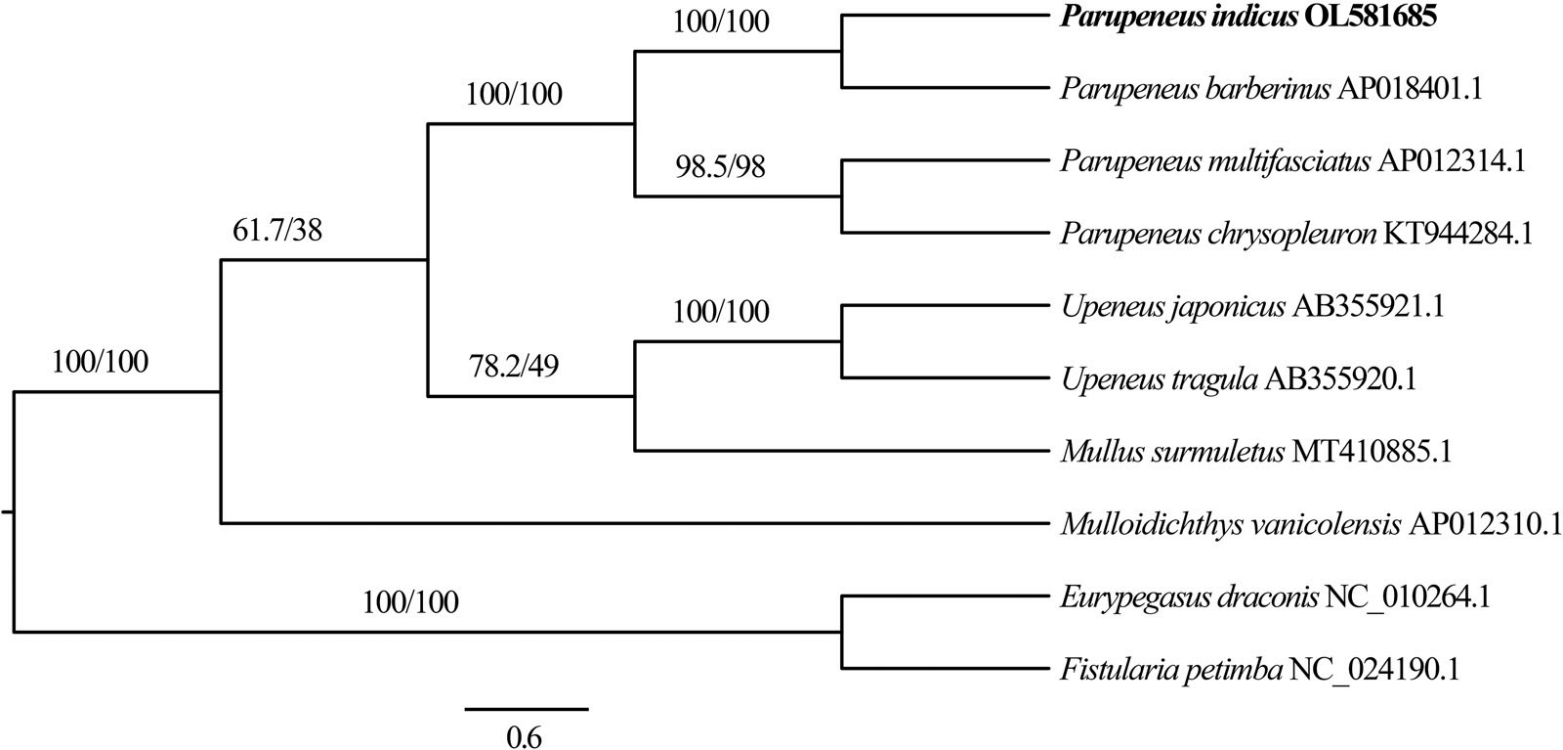
A phylogenetic tree of *Parupeneus indicus* and seven related species in the family Mullidae based on maximum likelihood analysis of the concatenated nucleotide sequences of 13 protein-coding genes. The numbers denoted at nodes are SH-like aLRT/bootstrap values.

## Data Availability

The genome sequence data that support the findings of this study are available at NCBI GenBank (https://www.ncbi.nlm.nih.gov) with the accession number OL581685. The associated BioProject, SRA, and Bio-Sample accession numbers are PRJNA800219, SRR17774844, and SAMN25236414, respectively.
